# Using deep learning–derived image features in radiologic time series to make personalised predictions: proof of concept in colonic transit data

**DOI:** 10.1007/s00330-023-09769-9

**Published:** 2023-06-07

**Authors:** Brendan S. Kelly, Prateek Mathur, Jan Plesniar, Aonghus Lawlor, Ronan P. Killeen

**Affiliations:** 1 Department of Radiology, St Vincent’s University Hospital, Dublin, Ireland; 2 Insight Centre for Data Analytics, UCD, Dublin, Ireland; 3 School of Medicine, University College Dublin, Dublin, Ireland

**Keywords:** Deep learning, Time series analysis, Radiology

## Abstract

**Objectives:**

Siamese neural networks (SNN) were used to classify the presence of radiopaque beads as part of a colonic transit time study (CTS). The SNN output was then used as a feature in a time series model to predict progression through a CTS.

**Methods:**

This retrospective study included all patients undergoing a CTS in a single institution from 2010 to 2020. Data were partitioned in an 80/20 Train/Test split. Deep learning models based on a SNN architecture were trained and tested to classify images according to the presence, absence, and number of radiopaque beads and to output the Euclidean distance between the feature representations of the input images. Time series models were used to predict the total duration of the study.

**Results:**

In total, 568 images of 229 patients (143, 62% female, mean age 57) patients were included. For the classification of the presence of beads, the best performing model (Siamese DenseNET trained with a contrastive loss with unfrozen weights) achieved an accuracy, precision, and recall of 0.988, 0.986, and 1. A Gaussian process regressor (GPR) trained on the outputs of the SNN outperformed both GPR using only the number of beads and basic statistical exponential curve fitting with MAE of 0.9 days compared to 2.3 and 6.3 days (*p* < 0.05) respectively.

**Conclusions:**

SNNs perform well at the identification of radiopaque beads in CTS. For time series prediction our methods were superior at identifying progression through the time series compared to statistical models, enabling more accurate personalised predictions.

**Clinical relevance statement:**

Our radiologic time series model has potential clinical application in use cases where change assessment is critical (e.g. nodule surveillance, cancer treatment response, and screening programmes) by quantifying change and using it to make more personalised predictions.

**Key Points:**

• *Time series methods have improved but application to radiology lags behind computer vision. Colonic transit studies are a simple radiologic time series measuring function through serial radiographs.*

• *We successfully employed a Siamese neural network (SNN) to compare between radiographs at different points in time and then used the output of SNN as a feature in a Gaussian process regression model to predict progression through the time series.*

• *This novel use of features derived from a neural network on medical imaging data to predict progression has potential clinical application in more complex use cases where change assessment is critical such as in oncologic imaging, monitoring for treatment response, and screening programmes.*

## Introduction

Colonic transit time studies (CTS) involve taking serial radiographs to track the passage of radiopaque beads through the digestive system [1, 2]. The study is over when all beads have passed through the tract, and can as such be framed as a time series problem. While there have been recent advances in time series methods, they have not been widely used in the medical imaging domain [3]. Furthermore, despite calls for AI models that can take prior imaging into account few models are designed to handle this type of data [4]. Siamese Neural Networks (SNN) however, have been used to measure the severity of change between radiologic studies [5]. SNNs employ two parallel neural networks that produce a feature vector based on the input. The difference in feature space between the two feature vectors can be calculated and used as a measure of the difference between the inputs. We used this distance as a feature in a Gaussian process regression model to predict progression through the time series.

### Colonic transit

Constipation is a problem that may affect up to 28% of the population and testing for constipation has been estimated at over 6.9 billion dollars in the USA [6]. As such it contributes significantly to the global care burden. Colonic transit time studies (CTS) involve taking serial radiographs to track the passage of radiopaque beads through the digestive system. CTS are used to diagnose and differentiate different causes of constipation [2]. The rate of disappearance of the beads in normal digestion can be described as exponential [1, 2]. Prediction of the endpoint of the time series has uses in cost reduction [1, 2] public health [7], and microbiome research [8] as well as potentially achieving earlier and more accurate diagnoses for patients. As such, we chose the CTS to experiment with current techniques capable of dealing with short time series data.

### Time series

A “time series” is a data representation that includes a temporal or sequential dimension; the study of time series involves understanding the consequences of dynamics and change over time [9]. There have been many developments in the field recently with the application of artificial intelligence techniques and new deep learning models such as LSTM. These include industrial [10] and more recently medical use cases [11]. Many of these use cases apply to expansive data sets with thousands of data points. In contrast, many clinical use cases do not have extensive data points in the time domain. We apply the methods of time series analysis to the problem of predicting the completion of a CTS study.

### Incorporating prior images into AI models

There has been a recent increase in the artificial intelligence and medical imaging literature. However, most of these studies focus on segmentation and classification for a narrow range of use cases [3]. Few studies delve into the issue of change between images, or predictions that change over time. For example, the largest open-access data depositories in medical imaging do not contain temporal information [12]. This is counterintuitive as one of the most important steps in clinical radiology is to compare a study to the previous one to analyse or quantify the degree of change. This has led to calls for models that can incorporate prior images. Indeed such models have been described as “essential to provide meaningful improvements” in the field [4]. While several methods have been proposed for the purpose of change detection based on AI [13] few have been tested in radiology [4]. While some studies do exist in this space [5, 14–17] considering recent developments in the wider machine learning literature [18] there are opportunities for advancement in the medical imaging field by using a wider range of techniques.

### Siamese neural networks

SNNs employ two parallel neural networks that produce a feature vector based on the input. The difference in feature space between the two feature vectors can be calculated and used as a measure of the difference between the inputs. This type of architecture was originally described as a method of one-shot image recognition [19]. A Facebook team also used a similar architecture as a method for facial recognition [20]. Recently, it has been applied to radiologic images in arthritis [5, 21] and Covid-19 [16].

### Progression

It is worthwhile to define what we are actually attempting to quantify with the SNN. Progression generally refers to a worsening of disease in the medical literature [22]. Time series methods have been employed in the medical literature to predict the progression of disease [22–25]. Progression through a time series, however, does not necessarily have negative connotations. As stated the SNN measures the distance in feature space between the two images. This distance has been used previously as a surrogate for disease severity [5, 21]. In the current study, we use distance as a measure of progression through the CTS (a time series). A larger difference between the images is used as a marker for a greater step (more progress) toward the end of the test.

### Aims

Our purpose was to use deep learning to identify radiopaque beads on radiographs as part of a CTS, to assess interval change between radiographs, and to predict progression through the study on a patient-specific basis.

### Contributions

In this paper we describe a novel use of neural network–derived features from medical imaging data: using the output of the SNN as a feature in a time series model to predict progression. Our methods could have potential clinical application in areas where change assessment is critical such as in oncologic imaging, monitoring for treatment response, and screening programmes. We also demonstrate a new method of incorporating prior images into AI models.

### Methods

This retrospective study was approved by our institution’s research ethics committee. The requirement for prospective consent was waived. This manuscript was prepared using the CLAIM checklist [26].

### Data

All patients undergoing a colonic transit study from 2010 to 2020 in a single tertiary referral centre were included.

Data were pre-processed in two steps. First, images were cropped to 93% of the original image to facilitate the removal of the anatomical side markers (left “L” or right “R”) as these were identified by the model in our pilot data. Then the cropped images were resized to 224 × 224.

Data were anonymised using a two-phase anonymisation technique, first integrated with our SIEMENS PACS system and second using a Python script modified from a previous HIPAA-compliant project [27].

Thirty-two patients had only one image included, and we included these for training but excluded them for per-patient analysis.

Ground truth was determined as the total number of beads present in the image. This was recorded from the radiology report and verified by two of the radiologist authors. No inter-reader variation was observed. No annotation tool was used. Time series data were retrieved from the timestamp in the DICOM metadata.

We split our data into train and test sets in a ratio of 80:20. Data were partitioned at the patient level. Our code is available on GitHub (https://github.com/pmath2012/ColonicTransit).

### Model

**Fig. 1:**
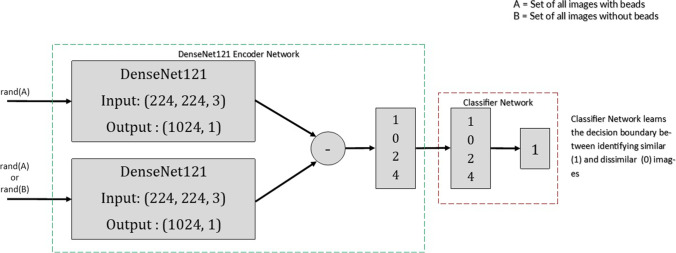
Title baseline network architecture

**Fig. 2:**
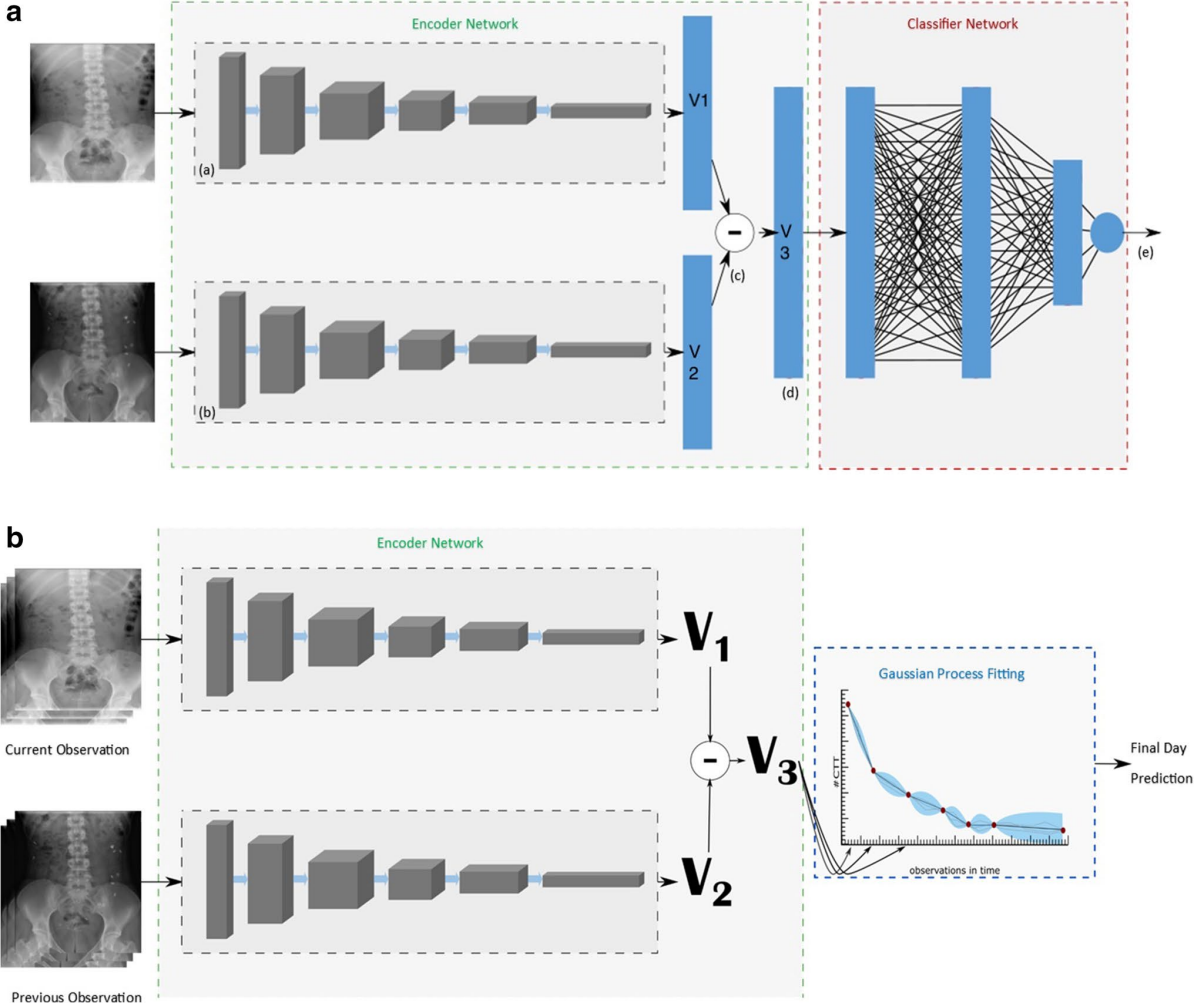
**a** Title: Siamese Classifier Network. Legend: Representation of the Siamese Classifier (a), (b) arms of the Siamese network containing Identical base Networks (DenseNet121) each having the same structure as Fig. 1 (c) Euclidean Distance between the produced feature vectors V1 and V2 (d) Representation of the Difference vector. The classifier network contains three densely connected layers with a normalisation layer (N) between layers one and two (2048 −> N −> 512 −>1) (e) Final prediction label. **b** Title: Gaussian Process Regressor. Legend: Representation of the Gaussian Process Model which uses the output of the Siamese network as features. The Euclidean distance in feature space (V3) between the feature vector of the current observation (V1) and previous observation (V2) is fed as a feature to the GPR

**Fig. 3:**
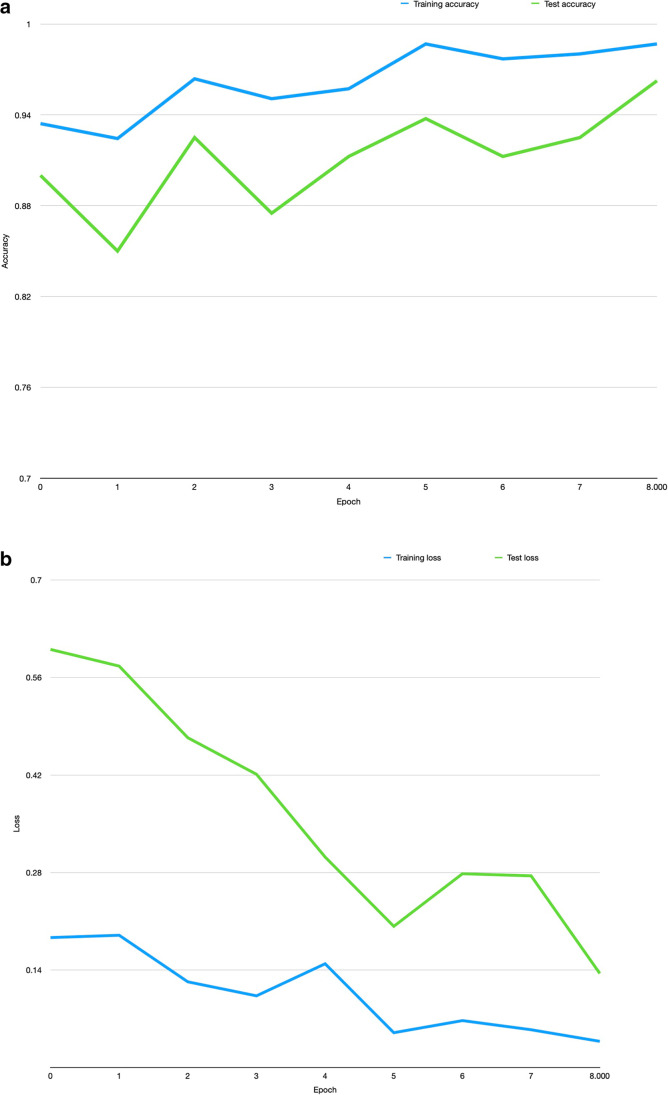
Accuracy (**a**) and loss (**b**) curves from the training log of the proposed model. Legend: Accuracy and training loss per epoch with early stopping to minimise overfitting. The training and validation loss do not converge

The base Network (Vanilla DenseNet network) of the DenseNet121 [28] was pre-trained using chest radiographs on the Stanford CheXpert Dataset [12]. Our pilot investigations used the vanilla DenseNet as a baseline model. The baseline architecture is illustrated in Fig. 1. It consists of a Siamese Neural Network (SNN) architecture with two parallel DenseNets. The DenseNet represents an encoder that learns a feature representation vector. We compute the distance between the two feature vectors and a final classifier (three densely connected layers with a normalisation layer (N) between layers one and two (2048 − > N -> 512 − >1) learns a decision boundary to differentiate *similar* from *dissimilar* images. Each arm of the SNN comprises identical base CNN networks. Thus, the Siamese encoder takes in two images and produces a representation of the difference vector of the inputs. This representation is fine-tuned using contrastive learning and then fed to a classification network (Fig. 2a). Accuracy and Loss per epoch are outlined in Fig. 3a and b.

**Fig. 4:**
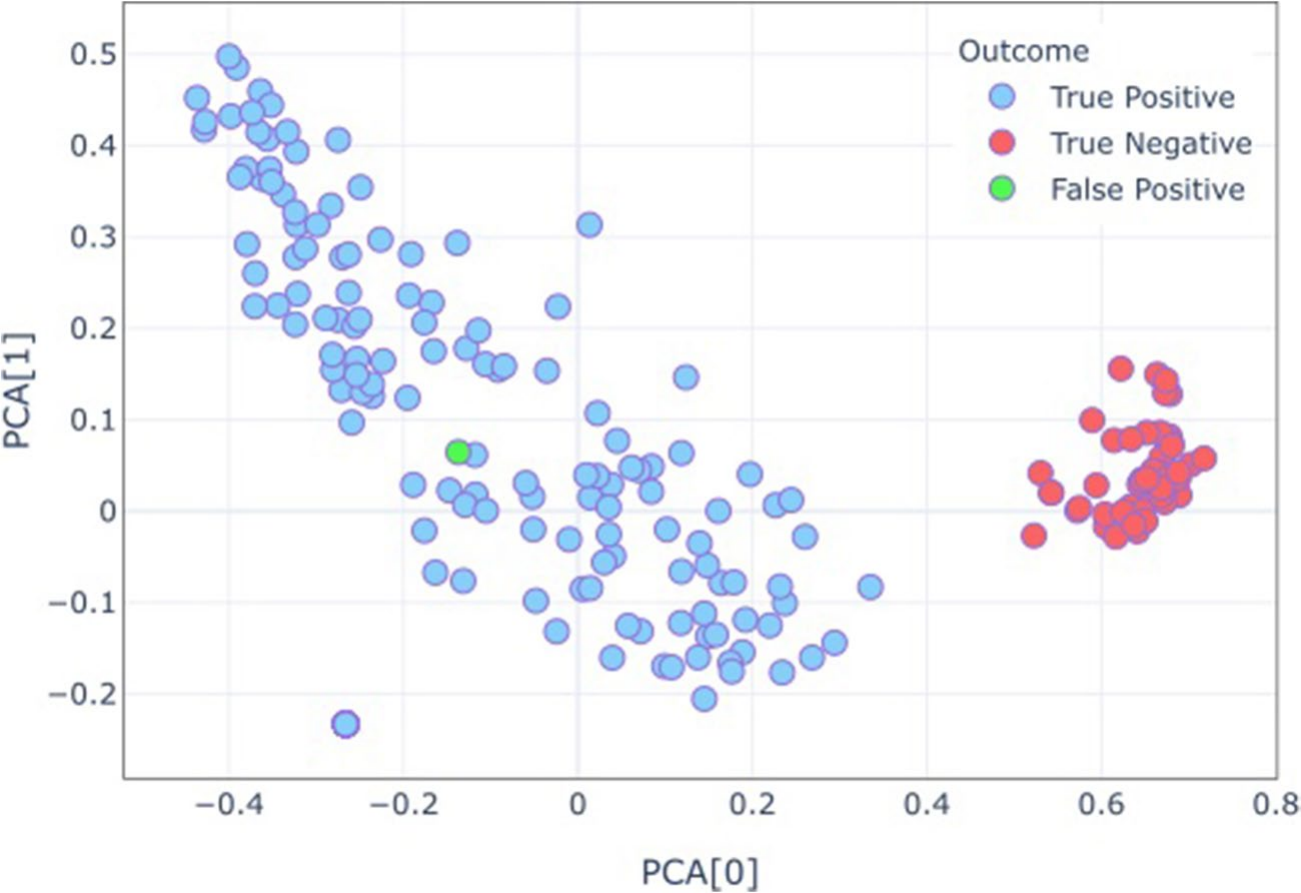
Title: PCA performed on the features extracted by the Siamese Network. Legend: PCA demonstrates that the Siamese network has learned features that accurately differentiate between classes. The true negatives are well separated from true positives in the feature space, allowing for some false positives

Dimensionality reduction using Principal Component Analysis (PCA) was applied on the extracted features to provide a visualisation of the decision boundary (Fig. 4).

For time series analysis we compared basic statistical curve fitting assuming exponential decay, to other state-of-the-art methods including XGBoost regression, long short term memory networks (LSTM), Gaussian process regressor (GPR), a LSTM trained on the outputs of the GPR and finally trained using the output of the SNN (distance measure) at each time point (Fig. 2B). Saliency maps for common false positive and false negative cases were produced and scrutinised.

### Training

Training of the SNN occurs in two steps: first, the SNN is trained using contrastive loss to learn the optimal representation of the difference vector. A projection network is appended to the Siamese Encoder and trained using contrastive loss [29]. The learning rate is set to 1e-4, the temperature of the contrastive loss is set to 0.08, and the Adam optimiser is used.

Following the contrastive training, the encoder network is then frozen, and only the classification network is trained. The learning rate is set to 1e-4 and Adam optimiser is used with binary cross-entropy loss. Early-stopping utilities are used to reduce the net training time.

The inputs to the SNN are generated using two strategies: Biased and Balanced. In the biased strategy, one input to the SNN is fixed to a single class. In the proposed strategy, one arm is set to always receive an image containing radio-opaque beads, referred to as a positive image. The second arm of the network receives a randomised choice between a positive or a negative image. In the balanced strategies, both arms can receive either positive or negative images. The label for classification is determined as positive (1) when both images are positive and negative (0) when either of the images is negative. The network and all computations are performed using Keras with TensorFlow backend on an NVIDIA GTX1080 Ti processor.

Data were randomly augmented at the level of each batch (batch size = 4) with a random application of width and or height shifts of 10% of the size of the image and/or horizontal flips.

For the time series portion, the baseline models were trained using the total number of remaining beads at each radiograph. The results of the Gaussian Process were used to train the LSTM (effectively stacking the GP and LSTM). Finally, a GPR was trained using the output of the Siamese Network (distance measure) at each time point. Hyperparameter optimisation was employed with a grid search for the XGBR and GPR.

### Evaluation

Our model was tested on an internally sourced but independent hold-out validation set representing 20% of the data. This 20% was not used for training. We evaluated our model using a combination of accuracy, area under the receiver operator analysis curve (AUC), precision-recall, and correlation. In experiments with balanced classes, we favoured accuracy as a metric [30]. The mean absolute error was used to evaluate the time series models. Saliency maps of false positives and false negatives (FP, FN) were interrogated for a better intuition of explainability and interpretability but were not used explicitly for localisation [31]. Statistical comparison between regression models was performed using Student’s T-test and by calculation of 95% confidence intervals.

## Results

### Demographics

In total, 570 radiographs of 230 patients were retrieved. Thirty-two patients had only one image and were not used in the time series analysis. One case was excluded during quality assurance as it was mislabelled as a CTS leaving 568 images of 229 patients (range 1–5 per patient) for primary analysis and 536 images of 197 patients for time series analysis. The median age of patients included was 57 years and 123 (54%) were female.

### Classification

Classification results for different models under different training conditions are given in Table 1. For binary classification of the presence or absence of beads, the best performing model (Table 1, bold) was Siamese DenseNET trained with a contrastive loss on balanced input data with unfrozen weights achieved an accuracy, precision, and recall of 0.988, 0.986, and 1 respectively. Figure 4 shows the results of PCA for the same model on the test set. The PCA is calculated for 2 components of 1024 features of one image.

**Table 1:** Title classification results

Training inputs & outputs	Models	Accuracy	AUC	Matthews Correlation Coefficient	Precision	Recall
*Random*	Vanilla DenseNet (Pretrained on Chexpert data)	0.981	0.98	0.95	0.98	0.98
Biased Per-patient Inputs	Siamese Densenet (Trained on Frozen Vanilla Densenet Encoder weights)	0.883	0.986	0.72	0.994	0.86
	Siamese Densenet (Trained on Unfrozen Vanilla Densenet Encoder weights)	0.97	0.991	0.91	0.995	0.967
	Siamese Densenet (Trained on Frozen Chexpert Densenet Encoder weights)	0.932	0.964	0.819	0.99	0.92
	Siamese Densenet (Trained on Unfrozen Chexpert Densenet Encoder weights )	0.958	0.983	0.882	0.995	0.953
Balanced Per-patient Inputs	Siamese Densenet (Trained on Frozen Vanilla Densenet Encoder weights)	0.966	0.997	0.9	0.995	0.962
	**Siamese Densenet (Trained on Unfrozen Vanilla Densenet Encoder weights)**	**0.988**	**0.945**	**0.964**	**0.986**	**1**
	Siamese Densenet (Trained on Frozen Chexpert Densenet Encoder weights)	0.895	0.958	0.744	0.99	0.87
	Siamese Densenet (Trained on Unfrozen Chexpert Densenet Encoder weights )	0.962	0.981	0.89	0.99	0.95

Legend Performance metrics for different models and conditions on the Test set. Best performing model in boldface

### Time series

**Fig. 5:**
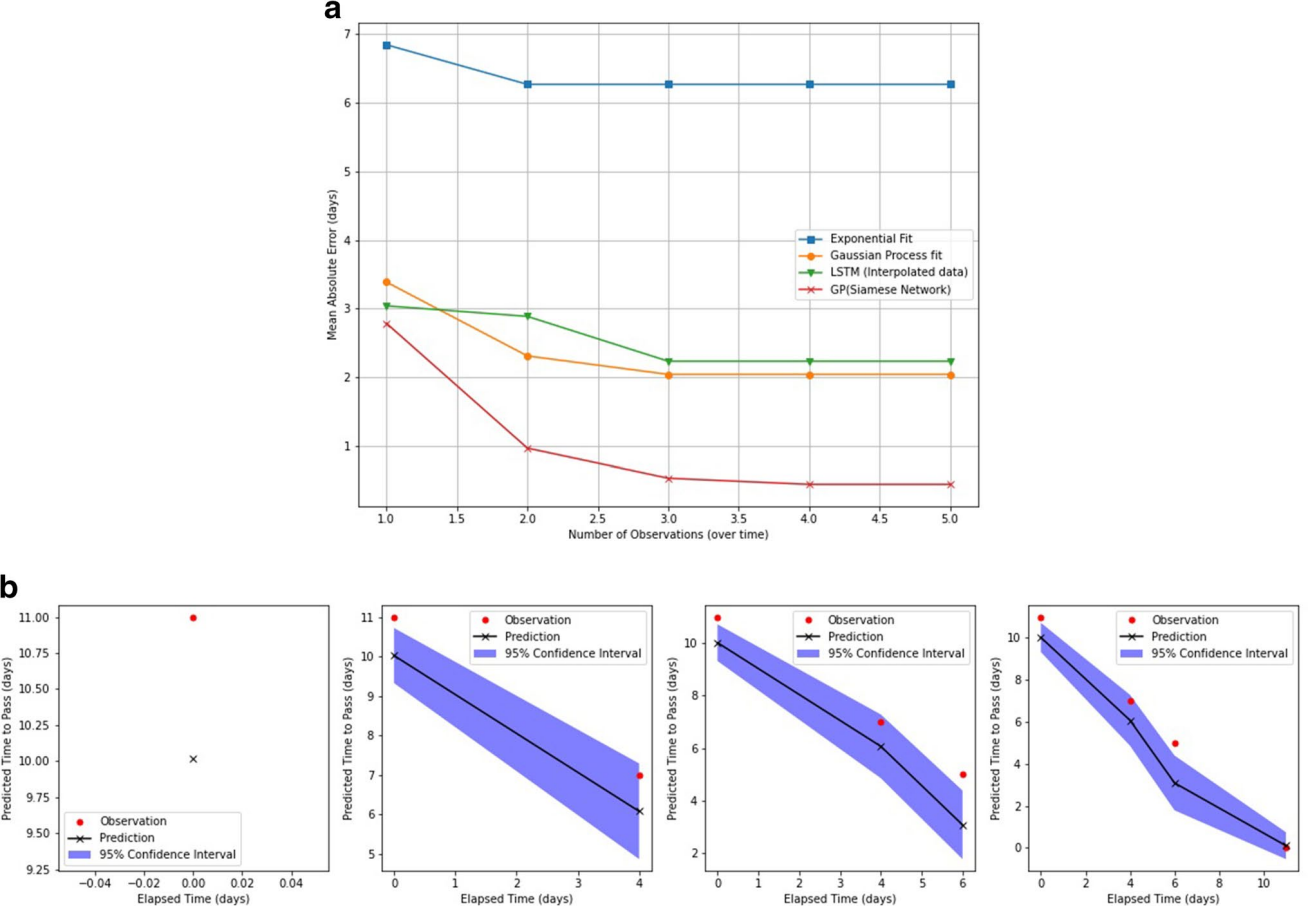
**a** Title: time series results. Legend: Mean absolute error (days) for prediction of the final day in the series for different models. **b** Title: Prediction and confidence interval for the GPR. Legend: Visualisation of how the prediction alters dynamically with each step in the time series.

For the prediction of the number of days until the last bead was passed different models we tested and compared using mean absolute error (MAE) (number of days). Performance is outlined in Fig. 5a.

After two radiographs (two points on the time series) Basic statistical curve fitting assuming exponential decay had a MAE of 6.3 days. Given the number of beads remaining, the MAE of an XGB regressor was 4.74 and the Gaussian process regressor (GPR) was 2.8. An LSTM trained on the outputs of a GPR achieved a MAE of 2.3 days. Finally, the MAE of a GPR trained on the output of the SNN (which uses information gleaned from the whole image not just the raw number of beads) was 0.96 days. This represented a large improvement on the other models and significantly outperformed basic statistical exponential curve fitting (*p* < 0.05).

Figure 5b shows how the confidence in predictions narrows with each step in the time series. The prediction improves with new information.

### Saliency maps

**Fig. 6:**
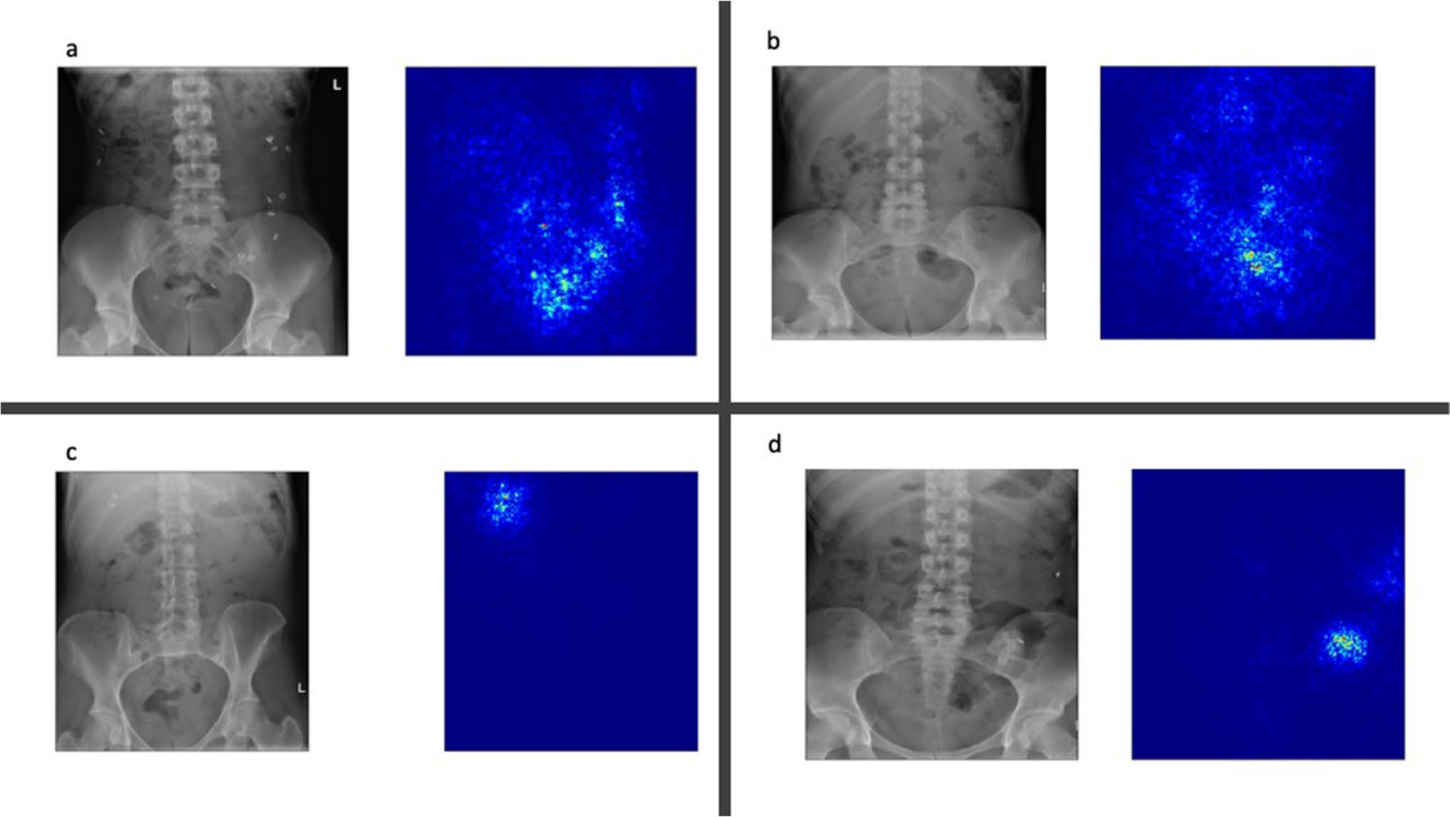
**a**–**d** Title: Saliency maps of an illustrative TP, TN, FP, and FN case. Legend: **a** shows a representative true positive image, **b** a representative true negative. **c** A false positive example, and (**d**) a false-negative case

Saliency maps were produced for all cases and reviewed by two radiologist authors. Representative images are shown in Fig. 6.

## Discussion

In this study, we built a deep learning model to identify radiopaque beads on the radiographs as part of a CTS. We employed lesser-used methods assessing interval change between radiographs using a SNN architecture. Also, we introduce an innovative application in the medical imaging domain by leveraging time series methods to predict the time to completion of the study on a patient-specific basis. Specifically, we used SNNs first to quantify the distance in feature space between two images of the same patient at different points in time and then used that distance measure to make a prediction about the future clinical course (progression) of that patient. Our methods could have potential clinical application in areas where change assessment is critical such as in oncologic imaging, monitoring for treatment response, and screening programmes. We also provide a novel method of incorporating prior images into AI models.

SNNs use two identical parallel models to learn feature representation vectors and their discriminatory power is derived from computing the distance between the feature vectors in the parallel models. While the use of SNNs in the radiology literature is rare, it is becoming more popular for change detection use cases such as in longitudinal pulmonary nodule progression [32], comparing current and prior mammograms [33], and computing pulmonary oedema severity as a “continuous-value” in chest radiography [34]. This is why we chose this method to discriminate between two similar radiographs with a different number of radiopaque beads. Its use in “one-shot” and “few shot”[34] learning methodologies shows the method is robust to small sample sizes and class imbalance, which is often the case for clinical applications such as CTS.

A “Time Series” is a data representation that includes a temporal or sequential dimension; the study of time series involves understanding the consequences of dynamics and change over time [9]. Recently time series methods have been combined with radiomics and other clinical and biomedical information and applied to the prediction of disease progression [35]. The CTS use case is suitable for trialling time series analysis methods for a number of reasons, including a clear start and end point and relatively unequivocal ground truth. Furthermore, the computer vision task is less complex with high contrast between the target (beads) and background (normal anatomy). The CTS uses single 2D images compared to multimodal 3D data from other modalities. These properties enabled us to focus on the time series aspect of the problem. Progression through the time series is presumed to be exponential with on average 16/20 beads remaining (1 day after ingestion), 8 (2 days after), 4 (3 days after), 2 (4 days after), and 1 (5 days after). As such our baseline method was statistical curve fitting using a simple exponential decay function and attempted to use machine learning methods to improve on that performance.

The XGBoost model showed some improvement over the baseline model but it is clear that the dynamic consequences of change are better captured by the GPR with illustrative examples given in Fig. 5. The GPR model allows us to extract a confidence interval for each prediction, and as we include more time steps we see the confidence intervals narrow, showing the predictions are steadily improving with an increasing number of samples. We investigated the use of an LSTM to predict progression with an ensemble method known as stacking to combine the outputs of our GPR model with an LSTM, and this results in a further improvement in performance.

While these methods achieved reasonable performance they are trained based only on the number of beads remaining in each image. There is much more information contained in the radiographs than just the number of beads. To capture this additional information we proceeded to use the output of the SNN to train a GPR. This model was our best-performing model for the prediction of “time to pass.” This method significantly outperformed the basic statistical curve fitting and can produce a personalised prediction that can dynamically change based on new information. Its success shows that the distance function output by the SNN captures meaningful information about the difference between two images over and above the change in a number of beads.

There are many potential applications of such a model beyond CTS. Much of clinical radiology is concerned with interval change and there are applications to areas such as pulmonary nodule surveillance, assessing for treatment response in oncologic imaging, following patients with multiple sclerosis, or as part of a screening programme. Increasing the range of tasks being worked on in the computer vision for medical imaging space and beginning to answer clinically relevant questions such as those mentioned above will require a shift in focus [4].

### Limitations

As this was a pilot study to examine the feasibility of new methods, we had a relatively small sample size and used a single-centre retrospective design. This will limit the generalisability of the model and also introduces a selection bias. However, we achieved high accuracy in the classification task and an increased sample size should make the time series aspect easier. As stated the task was relatively simple using 2D images with high contrast between the target and the background, this was to allow focus on the time series aspect of the study. It would be important for future studies to examine the use of these methods in advanced imaging such as MR and CT.

## Conclusion

Siamese Networks perform well at the identification of radiopaque beads in CTS. For time series prediction the use of deep learning improved performance over existing statistical models, enabling more accurate personalised prediction of progression. Such use of radiological image-derived features as inputs to a time series model, and the incorporation of prior images into AI models has potential application in many areas of clinical radiology beyond the CTS.

## Declarations

### Guarantor

The scientific guarantor of this publication is RP Killeen.

### Statistics and biometry

No complex statistical methods were necessary for this paper.

### Study subjects or cohorts overlap

NA

### Methodology

• retrospective

• experimental

• performed at one institution
